# Beyond the Physical: Weight Stigma and the Bariatric Patient Journey

**DOI:** 10.3390/jcm14020543

**Published:** 2025-01-16

**Authors:** Florin Vasile Mihaileanu, Mihaela Fadgyas Stanculete, Claudia Gherman, Vlad Dumitru Brata, Alexandru Marius Padureanu, Miruna Oana Dita, Daria Claudia Turtoi, Paolo Bottalico, Victor Incze, Bogdan Stancu

**Affiliations:** 1 Department of Surgery, County Hospital, “Iuliu Hatieganu” University of Medicine and Pharmacy, 400139 Cluj-Napoca, Romania; ms26rfl@yahoo.com (F.V.M.); gherman.claudia@umfcluj.ro (C.G.); bstancu7@yahoo.com (B.S.); 2 Department of Neurosciences, Discipline of Psychiatry and Pediatric Psychiatry, “Iuliu Hatieganu” University of Medicine and Pharmacy, 400394 Cluj-Napoca, Romania; 3 Department of Gastroenterology, Regional Institute of Gastroenterology and Hepatology “Prof. Dr. Octavian Fodor”, 400394 Cluj-Napoca, Romania; brata_vlad@yahoo.com; 4 Faculty of Medicine, “Iuliu Hatieganu” University of Medicine and Pharmacy, 400000 Cluj-Napoca, Romania; alexandru.padureanu@outlook.com (A.M.P.); miruna.dita@outlook.com (M.O.D.); vicincze@yahoo.com (V.I.); 5 Department of Radiology, County University Emergency Hospital, 400006 Cluj-Napoca, Romania; turtoidariaclaudia@gmail.com; 6 Department of Medical Sciences, University of Turin, 10126 Turin, Italy; paolo.bottalico@edu.unito.it

**Keywords:** obesity, bariatric surgery, weight stigma, obesity stigma, health-related quality of life

## Abstract

**Background:** Obesity represents a global epidemic associated with significant health risks, including diabetes, cardiovascular disease, and certain cancers. Management strategies have evolved from focusing solely on weight reduction to emphasizing overall health improvements and mitigating associated risks. **Methods:** This narrative review analyzed the existing peer-reviewed literature across databases such as PubMed, Scopus, EMBASE, the Cochrane Library, and Google Scholar to examine the outcomes of bariatric surgery and its interplay with weight stigma. The review incorporated data from clinical studies, systematic reviews, and meta-analyses, focusing on bariatric surgery and its psychological impact, as well as approaches to mitigate stigma in bariatric care. **Results:** Bariatric surgery has emerged as the most effective intervention for sustained weight loss and the resolution of obesity-related comorbidities. However, individuals undergoing surgery frequently encounter weight stigma, both pre- and postoperatively, which manifests through discriminatory healthcare interactions, societal biases, and internalized self-criticism. These factors contribute to anxiety, depression, diminished healthcare engagement, and weight regain, ultimately affecting the long-term surgical outcomes. **Conclusions:** Addressing weight stigma in bariatric care is critical to optimizing outcomes. Interventions such as preoperative counseling, postoperative psychological support, and multidisciplinary care can mitigate the psychological and societal burdens of stigma. This review explores the intricate relationships between obesity, bariatric surgery outcomes, weight stigma, and the health-related quality of life (HRQOL).

## 1. Introduction

Obesity, defined as a body mass index (BMI) exceeding 30 kg/m^2^, has emerged as a global health epidemic, contributing significantly to the burden of chronic diseases, such as diabetes, cardiovascular disorders, and certain malignancies [1,2,3,4].

The assertion that obesity is solely attributable to the food consumption quantity disregards substantial evidence indicating that the body weight is influenced by a complex and interrelated set of factors. These factors encompass biological elements such as genetics, epigenetics, psychological factors, and hormones, as well as social determinants of health including income, education, employment status, and access to healthcare. Furthermore, food industry practices, including marketing and lobbying, the availability of opportunities for physical activity, and experiences with other forms of stigmatization all play a critical role in shaping the body weight [5].

In the pursuit of effective interventions, bariatric surgery has gained prominence as a viable option for sustained weight loss and improved metabolic outcomes [6]. While the physiological benefits of bariatric surgery are well documented, an emerging body of literature suggests that weight stigma, prevalent in society, can significantly impact the overall success and psychological well-being of individuals undergoing these procedures [7].

Bariatric surgery encompasses a spectrum of procedures designed to induce weight loss by modifying the gastrointestinal tract anatomy and, in some cases, limiting nutrient absorption [4]. Common techniques include gastric bypass, sleeve gastrectomy, and adjustable gastric banding, each of which offers distinct mechanisms to achieve weight reduction [4]. The goals of obesity treatment have transformed from merely focusing on reducing the body mass index (BMI) to prioritizing improvements in the overall health quality and a reduction in obesity-related metabolic and cardiovascular comorbidities. Additionally, medical interventions that were once considered unsuitable for individuals with obesity, such as lower limb arthroplasty and organ transplantation, are now more accessible for treatment.

The physiological effects of these procedures are profound, leading to sustained weight loss, the resolution of comorbidities, and improvements in the overall quality of life [4]. However, despite the evident clinical benefits, the psychosocial aspects of bariatric surgery, particularly the impact of weight stigma, remain an area of growing concern.

Weight stigma refers to the negative social attitudes, beliefs, and discriminatory practices directed towards individuals based on their weight [8]. It manifests in various forms, including microaggressions (e.g., subtle jokes about weight), overt discrimination (e.g., employment bias), and internalized stigma (e.g., shame and self-blame) [8]. Weight stigma not only inflicts emotional distress but also hinders healthy behaviors and engagement with healthcare services, contributing to disparities in obesity-related health outcomes [9].

Weight stigma, the social evaluation and discrimination of individuals based on their weight, has significant clinical implications that extend beyond the emotional and psychological domains. Persistent exposure to negative attitudes and stereotypes can lead to internalized stigma, whereby individuals internalize and perpetuate detrimental narratives about themselves. This phenomenon can precipitate a cycle of shame, anxiety, and depression, impeding their ability to seek and adhere to medical care, adopt health-promoting behaviors, and achieve optimal well-being [6].

Clinically, weight stigma manifests in numerous ways and affects both physical and mental health outcomes. Research has consistently demonstrated that individuals experiencing weight stigma are at an elevated risk of developing chronic conditions, including cardiovascular disease, type 2 diabetes, and certain types of cancer. This increased risk is often attributed to the stress response triggered by stigma, which leads to physiological changes that enhance the vulnerability to disease [8]. Moreover, weight stigma can create barriers to accessing quality healthcare, as individuals may anticipate judgment or experience discriminatory treatment from providers, resulting in a delayed diagnosis and the inadequate management of health conditions [9,10].

Weight stigma can exert profound effects on the mental health of individuals undergoing bariatric surgery [11,12]. A fear of judgment and societal disapproval may contribute to heightened anxiety and depression, potentially influencing treatment adherence and follow-ups. Additionally, weight stigma may act as a barrier in seeking medical care, leading to delays in the recognition and management of postoperative complications. Understanding the psychological toll of weight stigma in the context of bariatric surgery is crucial for optimizing patient outcomes and tailoring holistic care [13,14,15].

The complex interrelationship between obesity surgery and weight stigma presents a multifaceted challenge. On one hand, bariatric surgery can provide individuals with a means to mitigate the pervasive weight stigma they encounter, potentially leading to enhanced self-esteem, an improved body image, and an increased quality of life. As patients achieve substantial weight loss, they may experience a reduction in discrimination and negative stereotypes, fostering a sense of empowerment and social acceptance. However, the process is not always linear. The decision to undergo surgery may itself be subject to stigmatization, with individuals facing criticism for pursuing what some perceive as an expedient approach to weight loss [16,17].

Furthermore, the postoperative period can present its own set of challenges. While physical transformations may lead to positive changes in social interactions, they can also precipitate new or intensified forms of stigma. Patients may contend with unrealistic expectations, pressure to maintain their weight loss, and persistent biases related to their surgical intervention. The intersection of these factors underscores the complexity of navigating weight stigma even after achieving significant weight loss through bariatric surgery [18,19,20].

Addressing this issue necessitates a multifaceted approach, including preoperative counseling, education, and ongoing support to equip individuals with the strategies to navigate societal biases and develop resilience in the face of potential stigma.

Acknowledging the impact of weight stigma on individuals undergoing bariatric surgery, there is an increasing emphasis on developing strategies to mitigate its effects. Interventions such as preoperative counseling, educational programs, and postoperative psychological support aim to equip individuals with coping mechanisms to navigate societal biases [21,22]. Furthermore, fostering a multidisciplinary approach involving healthcare providers, mental health professionals, and support groups is essential for establishing a comprehensive support system for patients undergoing bariatric interventions [23].

The effects of weight stigma persist long after bariatric surgery [24,25]. Even after significant weight loss from bariatric procedures, many individuals continue to face stigma and discrimination. This ongoing stigma can undermine the anticipated benefits of surgery and negatively impact the quality of life [24]. Addressing weight stigma is a public health priority, with experts arguing that stigmatizing individuals for their weight is not an effective public health strategy [25,26]. Instead, weight stigma should be recognized as a social justice issue that requires intervention to improve the health and well-being of individuals affected by obesity.

The complexities surrounding weight regain and stigma after bariatric surgery necessitate a narrative review to provide a comprehensive synthesis of the existing evidence. Unlike systematic reviews, which focus on quantifiable outcomes, a narrative review allows for the integration of diverse perspectives, including qualitative, psychosocial, and clinical dimensions. This approach is particularly valuable in understanding multifactorial issues such as weight stigma, which intersects with behavioral, societal, and healthcare dynamics. By consolidating insights from varied methodologies and disciplines, this review aims to identify the underlying causes of weight regain, explore its impact on the health-related quality of life (HRQOL), and highlight the role of stigma as a barrier to optimal outcomes. The findings from this narrative review can inform public health strategies, guide clinical interventions, and shape policy decisions to improve the care and support provided to individuals undergoing bariatric surgery.

## 2. Materials and Methods

### 2.1. Data Sources and Search Strategy

**Figure 1:**
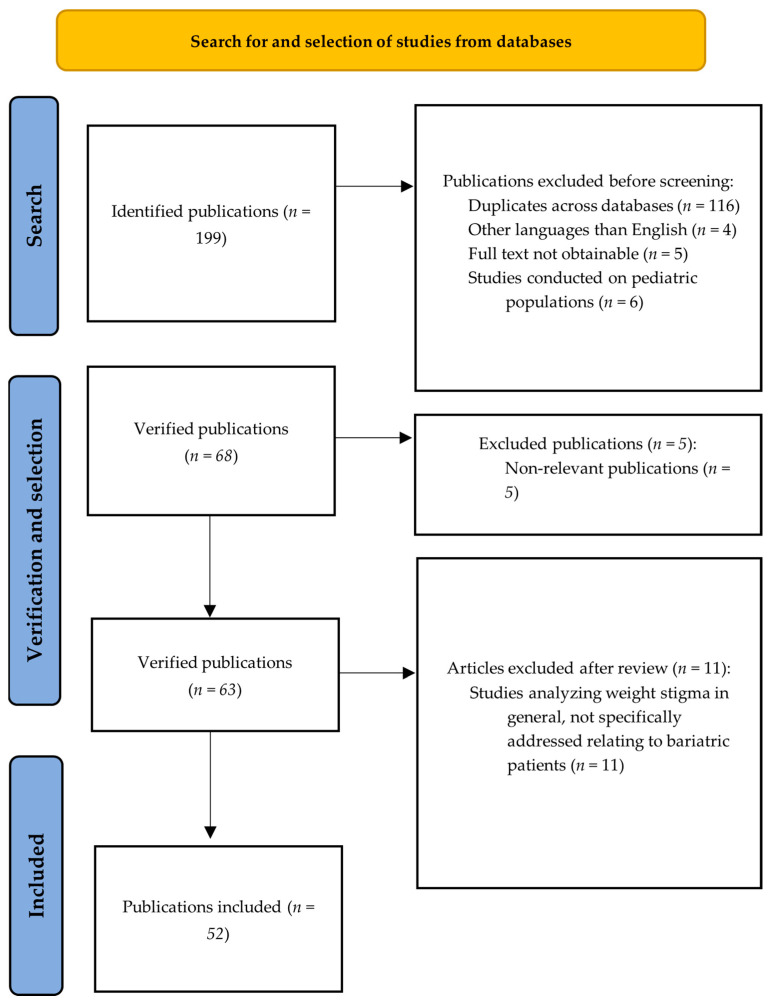
Study selection for this narrative review.

Electronic databases such as PubMed, EMBASE, Scopus, the Cochrane Library, and Google Scholar were searched from their inception until 30 December 2024 to identify potential studies. The search string entered in PubMed was (“bariatric surgery” OR “weight loss surgery”) AND (“obesity” OR “obesity-related outcomes”) AND (“weight stigma” OR “weight discrimination” OR “social stigma”) AND (“health-related quality of life” OR “HRQOL”) AND (“psychological outcomes” OR “mental health”) AND (“weight regain” OR “postoperative weight regain”). Furthermore, the literature selection process is shown in Figure 1.

### 2.2. Study Selection and Eligibility Criteria

Observational and interventional studies, as well as systematic reviews and meta-analyses, were included in this systematic review. Studies were included if they focused on bariatric surgery and its impact on obesity-related outcomes or the quality of life and addressed weight stigma in the context of bariatric care. Studies published in other languages than English, abstracts without the full text or with the paper unavailable, editorials, and preclinical studies, as well as studies conducted on pediatric patients, were excluded from this review.

## 3. Weight Stigma and Its Role in Weight Regain After Bariatric Surgery

Weight stigma exerts significant psychological and behavioral effects on individuals who have undergone bariatric surgery, contributing to weight regain and complicating the long-term outcomes.

Weight bias internalization, the societal stigma surrounding obesity, and prejudice regarding bariatric surgery itself are critical factors that exacerbate feelings of social isolation and disordered eating behaviors. Patients who internalize such biases often experience reduced motivation to engage in physical activity, further hindering their ability to maintain weight loss over time [27]. These psychological barriers make it challenging for patients to adhere to the lifestyle changes required for sustained weight management. Additionally, the shame associated with weight regain frequently deters patients from attending follow-up appointments, complicating efforts to monitor and address postoperative outcomes effectively [27].

Despite the challenges posed by weight regain, some studies highlight that bariatric surgery continues to offer certain benefits even when weight regain occurs. For instance, a study by Tolvanen et al. found that while participants experienced emotional distress—such as hopelessness, discouragement, shame, and frustration—related to weight regain, they also reported enduring positive effects from the surgery, such as improvements in health and initial weight loss benefits [28]. This dichotomy underscores the complex interplay between the physical and psychosocial outcomes of bariatric surgery.

## 4. The Role of Postoperative Lifestyle Changes and Familial Support in Bariatric Surgery Outcomes

Family dynamics are a critical factor in the prevalence and impact of weight stigma, significantly influencing individuals’ psychosocial health and behaviors. Research has highlighted that family-based weight stigma is widespread across various cultural contexts, with immediate family members often acting as primary sources of weight-related criticism, judgment, and mistreatment [29]. Notably, mothers (49–62%), spouses or romantic partners (40–57%), and fathers (35–48%) are frequently identified as key contributors to familial weight stigma, which is associated with poorer psychosocial health outcomes across multiple countries [29].

This stigma manifests in various ways, including negative assumptions about maternal or fetal health during pregnancy, comparisons to unattainable or idealized body types, and verbal comments that promote self-judgment and shame [30]. While close interpersonal relationships, such as those with family, are typically protective factors for mental health and facilitators of healthy behaviors, they can paradoxically become sources of weight stigma [30]. This duality underscores the complex and nuanced role of family dynamics in shaping the experience of weight stigma.

The internalization of familial weight stigma has far-reaching consequences, including the adoption of maladaptive eating behaviors, reduced physical activity, and heightened psychological stress. These behaviors not only exacerbate weight gain and obesity but also perpetuate a cycle of negative health outcomes [31,32]. Weight stigma within families can undermine efforts to address and prevent obesity by creating environments that discourage positive health changes and promote feelings of failure and hopelessness [32].

Family support can act as both a risk and a protective factor in maintaining weight loss following bariatric surgery. Research by Ugarte et al. highlights the complex dynamics of familial influence, revealing that “family identity”—a strong sense of connection and belonging within the family—was inversely associated with weight regain [33]. This suggests that patients with robust family ties may benefit from a supportive environment that facilitates sustained weight management.

Additionally, the study identified self-efficacy and the locus of control as significant psychological factors distinguishing between patients who successfully maintained their weight loss and those who regained at least 15% of their lost weight. High self-efficacy, reflecting confidence in one’s ability to adhere to postoperative guidelines, and an internal locus of control, emphasizing personal responsibility over external circumstances, were critical determinants of long-term success. These findings underscore the importance of fostering both supportive family dynamics and individual psychological resilience to optimize bariatric surgery outcomes [33].

Postoperative lifestyle changes, such as dietary modifications and increased physical activity, are critical for optimizing outcomes after bariatric surgery. These changes play a pivotal role in managing chronic conditions, including diabetes, cardiovascular diseases, and obesity, which are often associated with severe health complications [34]. However, the successful adoption of these lifestyle modifications requires significant adjustments, not only by the patient but also by their family, underscoring the importance of familial involvement in long-term success [35].

For bariatric surgery patients, the early incorporation of exercise has been shown to yield numerous benefits, including improved physical performance, enhanced glycemic control, reduced anesthesia-related risks, and faster recovery [36]. Moreover, systematic exercise after surgery is associated with an improved quality of life, enhanced insulin sensitivity, additional weight loss, and a better body composition [37]. Notably, the synergistic effect of combining diet changes and physical activity has a more pronounced impact on the BMI and health outcomes than either factor alone, highlighting the importance of a holistic approach to postoperative care [38].

However, the successful implementation of these lifestyle changes often hinges on familial support and education. Studies indicate that while there is widespread acknowledgment of the importance of dietary patterns and healthy lifestyle modifications among individuals with type 2 diabetes, the actual practice of these behaviors remains inadequate [38]. This suggests that family members can play a crucial role in facilitating or hindering adherence to these changes. For instance, family education on the demands and implications of dietary and physical activity adjustments may foster a supportive environment that enables the patient to maintain their postoperative regimen. Conversely, a lack of understanding or conflicting attitudes within the family may obstruct these efforts and undermine the long-term outcomes.

The importance of nutritional intake and behavioral modifications extends beyond the patient, influencing broader family dynamics. For example, during pregnancy, nutritional behaviors significantly impact maternal and fetal health, emphasizing the interconnectedness of individual and familial health behaviors [39]. Similarly, in the context of bariatric surgery, familial adjustments to lifestyle changes may improve not only the patient’s health outcomes but also the overall health quality of the family.

The role of family support in bariatric surgery is multifaceted, shaped by cultural, demographic, and socioeconomic factors that significantly influence patient outcomes. Research has highlighted that the family dynamics and functioning are critical in obesity management, particularly among pediatric and adolescent populations. Poor family functioning is closely linked to childhood obesity, which may persist into adolescence and impact the success of surgical interventions. Active involvement and support from family members are essential for fostering the lifestyle changes required for sustained weight loss post-surgery [40].

Cultural and socioeconomic factors also influence family dynamics and support systems. Economic strain and interparental conflict, common in low-income households, negatively affect the family’s well-being and can exacerbate obesity-related challenges [41]. Family communication patterns, crucial for successful obesity treatment, are often shaped by diverse cultural backgrounds and family structures, which influence the effectiveness of support mechanisms [42]. Evidence further suggests that high-functioning family systems correlate with better health outcomes, emphasizing the importance of positive family interactions in promoting healthier behaviors and long-term success [43].

Demographic diversity plays a crucial role in access to bariatric surgery and family support. Hispanic and African American patients are less likely to be referred for bariatric surgery than their white counterparts, often due to systemic barriers and cultural attitudes toward obesity [44,45]. Family support is a key predictor of surgery acceptance, particularly in communities where cultural beliefs may stigmatize obesity and surgical interventions. Tang et al. demonstrated that familial encouragement significantly improves acceptance rates, illustrating the importance of engaging families in treatment strategies [46].

Among Asian populations, family support reveals a complex interplay of cultural and demographic factors. Strong family ties, a hallmark of many Asian cultures, provide emotional and practical support but can also pose challenges. Familial expectations regarding health and weight may put pressure on patients, complicating their decision-making process [46,47]. In South Asian communities, patriarchal family structures can hinder individual autonomy, particularly for women, limiting their ability to access necessary medical care [48]. Additionally, the stigma associated with obesity often exacerbates these challenges, as societal and familial attitudes may prioritize the collective reputation over individual health needs [49].

Acculturation adds further complexity, particularly among Asian immigrants, whose experiences with family support evolve in new cultural environments. Wu et al. found that acculturation levels and weight stigma influence health outcomes, with immigrant families often facing stressors such as the absence of extended family networks [50]. These barriers can complicate the bariatric surgery journey, particularly regarding recovery and adherence to lifestyle changes [51].

Stigma and cultural perceptions of weight also intersect with eating behaviors. For instance, weight stigma contributes to disordered eating, such as binge eating, among Asian Americans, highlighting how cultural attitudes can significantly affect mental health and family dynamics [49]. Addressing these issues requires culturally sensitive approaches that engage families as partners in care and acknowledge the unique challenges faced by different populations.

In conclusion, family support is deeply intertwined with cultural, demographic, and socioeconomic factors in the context of bariatric surgery. Understanding the interplay between these elements is critical for healthcare providers to design effective, culturally adaptive interventions that address both medical and psychosocial aspects of obesity treatment. Future research should continue to explore these intersections to ensure equitable and successful outcomes across diverse populations. Addressing weight stigma within family contexts is essential for improving psychosocial health and supporting effective weight management strategies. Interventions should aim to educate family members on distinguishing between supportive communication and stigmatizing behaviors to foster a more empathetic and constructive environment. Future research must focus on designing and evaluating such interventions to mitigate the negative impacts of familial weight stigma and enhance the overall health outcome.

## 5. The Psychological Dimensions of Weight Regain After Bariatric Surgery

Psychological factors significantly influence the likelihood of weight regain following bariatric surgery. Several studies have identified key psychological variables that contribute to this phenomenon, highlighting the importance of mental health support in postoperative care.

Low self-efficacy and an external locus of control are among the strongest predictors of weight regain. Patients who successfully maintained their weight loss demonstrated higher levels of self-efficacy and an internal locus of control compared to those who experienced weight regain, suggesting that confidence in one’s ability to adhere to lifestyle changes and a sense of personal responsibility are critical for long-term success [33].

Depression, anxiety, emotional eating, and binge eating are also prevalent among individuals experiencing weight regain. These conditions often create a cycle of maladaptive behaviors that undermine efforts to maintain weight loss [52]. Interestingly, the presence of multiple psychiatric conditions has been implicated in weight regain. Although no single psychological condition prior to surgery consistently predicts weight loss outcomes, current diagnoses of binge-eating disorder, bulimia nervosa, alcohol abuse or dependence, and obsessive–compulsive disorder are significantly associated with weight regain [53,54].

Eating-related psychopathologies such as binge eating, disinhibition, heightened hunger levels, and impulsivity traits further exacerbate the risk of postoperative weight regain. These behaviors and traits often interact with underlying psychological distress, creating a complex interplay of factors that challenge long-term weight maintenance [54].

Difficulties in emotional regulation, lower self-efficacy, and food addiction symptoms have been identified as significant contributors to weight regain [55]. Patients with greater difficulties in emotional regulation were more likely to experience weight regain, while those with higher self-efficacy demonstrated greater success in sustaining weight loss [56]. Furthermore, the presence of food addiction symptoms was negatively associated with weight loss maintenance, emphasizing the importance of addressing these behaviors in postoperative care [56].

Interestingly, positive psychological factors are equally influential in promoting weight maintenance. Life satisfaction, conscientiousness, and positive affect were significantly associated with weight loss, with these factors being markedly higher among individuals in the highest weight loss quartile [56]. This suggests that fostering positive psychological states may play a complementary role in mitigating negative factors in preventing weight regain.

In conclusion, psychological factors—including low self-efficacy, maladaptive eating behaviors, and mental health disorders—are strongly associated with weight regain after bariatric surgery. Addressing these issues through targeted psychological support and interventions is crucial for preventing weight regain and enhancing the long-term surgical outcomes. A multidisciplinary approach integrating mental health professionals into bariatric care teams could provide the ongoing support necessary to mitigate these risks and optimize patient success.

## 6. Barriers and Opportunities in Postoperative Care

Postoperative care for bariatric surgery patients faces several challenges that can impede long-term success yet also offers numerous opportunities for improvement. A significant barrier is patient attrition from follow-up care, with certain demographic groups, including self-pay patients, males, and racial/ethnic minorities, being more likely to disengage within the first year following surgery [57]. Factors such as the financial burden, time constraints, and geographic limitations further contribute to difficulties in maintaining long-term engagement with care [58].

Primary care physicians (PCPs) play a critical role in the postoperative management of bariatric surgery patients. However, many PCPs report a lack of confidence in providing this care. Although PCPs are generally supportive of bariatric surgery and acknowledge its benefits, fewer than half feel adequately prepared to manage patients postoperatively, citing unfamiliarity with bariatric-specific follow-up requirements as a significant barrier [59,60]. This lack of confidence and knowledge among providers underscores the need for enhanced training and collaboration between bariatric centers and PCPs.

Addressing these challenges presents a range of opportunities to optimize postoperative care. Remote assessments and behavioral interventions have been shown to mitigate barriers related to accessibility, such as geographic and time constraints, while also improving patient outcomes [58]. Enhanced recovery after surgery (ERAS) protocols and standardized postoperative pathways could further streamline care delivery and minimize complications [61,62]. Additionally, patients have expressed a strong need for extended follow-up care, particularly psychological support, highlighting the importance of integrating mental health services into postoperative programs [63].

Improving communication between bariatric surgery centers and PCPs is another critical strategy for enhancing care. Providing PCPs with targeted training in bariatric patient management and addressing patient expectations and health literacy could facilitate better care continuity [64]. Education programs aimed at healthcare providers and trainees could also increase referral rates and access to bariatric surgery, ensuring that more patients benefit from this life-saving intervention [60].

In conclusion, while postoperative care after bariatric surgery presents significant challenges, targeted strategies such as remote interventions, standardized care protocols, enhanced psychological support, and improved provider training offer promising avenues for improvement. By addressing these barriers and leveraging these opportunities, the long-term outcomes and overall quality of care for bariatric surgery patients could be significantly improved.

## 7. Addressing Stigma Through Interventions

Weight stigma has been identified as a significant factor influencing mental health outcomes following bariatric surgery. Research has indicated that experiences of weight stigma are independently associated with increased symptoms of depression, anxiety, and binge eating after surgery, even when accounting for variables such as weight, demographic factors, and the pre-surgery mental health status [65]. This underscores the enduring impact of stigma and discrimination on patients’ psychological well-being postoperatively.

Conversely, bariatric surgery has been shown to enhance the quality of life and body image. Studies report significant improvements in the health-related and weight-related quality of life, as well as body image, within the initial months following surgery, with these benefits largely sustained into the second postoperative year [66]. This suggests that, despite the ongoing challenges posed by weight stigma, bariatric surgery can positively influence self-perception and the overall quality of life.

A series of studies has shown that interventions reducing weight bias before and after surgery have significant potential for mitigating the negative effects of weight stigma, improving the outcomes [67,68]. Furthermore, psychosocial interventions have seen a rise in popularity during recent years, being used to help patients to prepare for and adjust to the changes following bariatric surgery. Cognitive behavioral therapy has been shown to be the most beneficial in improving eating behaviors, as well as psychological functioning [69], which could, in turn, reduce the associated stigma for bariatric patients. These psychosocial interventions are most beneficial when initiated early during postoperative care in order to reduce the occurrence of pathological eating behaviors and weight regain [69].

### 7.1. Preoperative Counseling

Studies have suggested that patients with a history of psychiatric treatment or substance abuse counseling may achieve more favorable weight loss outcomes after bariatric surgery [65]. This finding challenges the assumption that such histories should be contraindications for surgery and underscores the potential benefits of addressing mental health issues prior to the procedure. By proactively managing psychiatric conditions, patients may develop coping mechanisms and resilience that support their postoperative success.

To address these complexities, comprehensive preoperative counseling is crucial. Such counseling should educate patients about realistic surgical outcomes, the behavioral changes required postoperatively, and strategies for managing stigma and psychological challenges [66]. Interventions targeting weight stigma at both the societal and individual levels are also essential for optimizing the long-term quality of life and related outcomes [63]. These measures could empower patients to navigate the psychological hurdles associated with bariatric surgery and enhance their postoperative experience.

Incorporating these elements into preoperative care will ensure that healthcare providers adequately prepare patients for the mental health and behavioral demands of bariatric surgery. This holistic approach will not only improve patient readiness but may also lead to better long-term surgical outcomes.

### 7.2. Provider Education

Provider education is pivotal in mitigating stigma associated with bariatric surgery and enhancing patient outcomes. Both internalized weight stigma and bias from healthcare professionals have been shown to negatively affect dietary adherence and the overall success of bariatric interventions [70]. To address these challenges, targeted education for healthcare providers is critical, focusing on the following key areas:

(1) Understanding the Impact of Weight Stigma:

Providers must recognize that weight stigma, including biases surrounding bariatric procedures, contributes to social isolation, disordered eating, and diminished motivation for physical activity [23]. An increased awareness of these issues could help foster empathy and a more patient-centered approach in clinical care.

(2) Recognizing the Importance of Long-Term Follow-Ups:

A long-term follow-up is crucial for sustaining behavioral changes and supporting dietary adherence. Providers need to prioritize ongoing care to help patients navigate challenges such as weight regain and motivational lapses [71]. Extending follow-up care will ensure that patients receive the support necessary for long-term success.

(3) Improving Communication and Counseling Skills:

Providers should be equipped with skills to discuss weight loss options confidently and effectively with potential surgical candidates. This includes knowledge of patient qualification criteria, streamlined communication strategies, and reassurance of the safety and efficacy of bariatric surgery [72].

(4) Addressing Misconceptions About Bariatric Surgery:

Educational initiatives should focus on dispelling myths, such as exaggerated perceptions of surgical risks compared to the benefits. Correcting these misconceptions among providers could reduce barriers to referrals and improve patient access to appropriate care [73,74].

(5) Promoting Patient-Centered Care:

Training providers in therapeutic patient education could empower individuals to actively participate in their care. This patient-centered approach will not only reduce stigma but will also encourage sustained engagement in health-promoting behaviors, improving the overall outcomes [73,75].

### 7.3. Policy Advocacy

Policy advocacy aimed at reducing stigma after bariatric surgery must address both societal- and individual-level factors to effectively improve the experiences and outcomes of patients. Despite the significant weight loss achieved through bariatric surgery, weight-related stigma continues to persist, negatively impacting patients’ mental health, quality of life, and ability to adhere to postoperative regimens [27,65].

(1) Societal-Level Interventions

Weight stigma in societal contexts remains pervasive, often fueled by misconceptions about obesity and weight management. Patients who undergo bariatric surgery frequently face biases that frame surgical intervention as a “shortcut” to weight loss, undermining their achievements and reinforcing stigma [72]. This societal bias can lead to social isolation, disordered eating behaviors, and reduced motivation for physical activity [27]. To combat these challenges, policies should achieve the following:

(a) Raise public awareness: Implement public health campaigns to educate the general population on the complex, multifactorial causes of obesity, including genetic, environmental, and psychological factors. Such efforts could challenge the simplistic narratives often associated with obesity and bariatric surgery.
(b) Promote anti-stigma initiatives: Develop programs that actively combat weight stigma in workplaces, schools, and the media. These initiatives should emphasize respect and inclusivity, encouraging environments that support individuals in their weight management journeys.
(c) Regulate healthcare practices: Establish guidelines to ensure weight-neutral approaches in healthcare settings. For instance, standardized communication protocols could prevent stigmatizing language and attitudes when interacting with bariatric surgery patients.

(2) Healthcare-Specific Policies

Weight stigma within healthcare settings is particularly detrimental, as it can erode patients’ trust and engagement with care. Policies should focus on training healthcare providers to recognize and address both overt and implicit weight biases. Research has shown that patients who experience stigma from healthcare professionals are less likely to adhere to dietary recommendations, further complicating their recovery [13].

Provider Education: Policies must mandate comprehensive training for healthcare professionals on the psychological and physiological aspects of obesity, the effectiveness of bariatric surgery, and the detrimental impact of stigma. Emphasis should be placed on fostering patient-centered care that prioritizes empathy and respect [76].

The Integration of Psychological Support: Weight stigma, including internalized bias, is a predictor of poor adherence to dietary guidelines and a diminished quality of life post-surgery [13]. Policies should ensure that psychological support is an integral component of post-surgical care plans, offering patients tools to manage internalized stigma and develop coping strategies [76,77].

Extended Follow-Up Care: Policies should advocate for long-term follow-up programs that include behavioral counseling and nutritional guidance, as these interventions could address the psychological and behavioral challenges associated with stigma and weight regain [76].

(3) Individual-Level Interventions

While systemic changes are essential, addressing the internalized stigma that many bariatric surgery patients experience is equally critical. Internalized stigma, or the acceptance of societal stereotypes about obesity, can perpetuate feelings of shame and self-blame, undermining long-term success [13,76]. Effective policies should achieve the following:

Encourage Therapeutic Interventions: Support funding for evidence-based interventions, such as cognitive behavioral therapy (CBT), to help patients challenge negative self-perceptions and build resilience against societal biases.

Promote Peer Support Groups: Peer-led initiatives can provide patients with a sense of community and shared understanding, helping them navigate the psychological challenges of postoperative life.

Effective policy advocacy for reducing stigma after bariatric surgery requires a comprehensive approach that addresses both societal biases and the internalized stigma experienced by patients [78]. Key strategies include educating healthcare professionals, integrating psychological support into care plans, and fostering public awareness about the complexity of obesity and the legitimacy of bariatric surgery as a medical intervention [67,78]. By addressing these factors, we can enhance the long-term outcomes and quality of life for bariatric surgery patients, creating a more equitable and supportive healthcare environment.

## 8. Discussion

The findings of this review highlight the profound impact of weight stigma on the psychological, behavioral, and physical outcomes of patients undergoing bariatric surgery. Weight stigma, both external and internalized, remains a pervasive issue, contributing to challenges such as social isolation, disordered eating, and reduced adherence to lifestyle modifications. These factors are closely linked to weight regain, a phenomenon that undermines the long-term benefits of bariatric surgery despite the procedure’s potential to improve the health-related quality of life.

The role of family dynamics emerged as a double-edged sword in this context. While familial support can enhance adherence to postoperative guidelines and foster psychological resilience, weight-related criticism from family members often exacerbates feelings of shame and self-judgment. Such dynamics underscore the need for targeted interventions that educate families about the distinction between supportive communication and stigmatizing behavior. Equipping families with the tools to provide constructive support can create an environment conducive to sustained weight management.

Psychological factors, including self-efficacy, the locus of control, and emotional regulation, were identified as significant predictors of postoperative outcomes. Patients with higher self-efficacy and an internal locus of control exhibited better adherence to dietary and physical activity guidelines, while difficulties in emotional regulation and food addiction symptoms were associated with a greater likelihood of weight regain. Interestingly, positive psychological factors such as life satisfaction and conscientiousness were found to complement these outcomes, suggesting the need for a dual approach that addresses both the mitigation of negative influences and the promotion of positive psychological states.

From a systemic perspective, barriers in postoperative care, including patient attrition and provider knowledge gaps, remain critical challenges. Limited follow-up care and a lack of confidence among primary care providers in managing bariatric surgery patients hinder optimal long-term outcomes. Remote interventions, enhanced recovery pathways, and provider education emerged as promising strategies to address these challenges. Policies that integrate psychological support and extend the follow-up care are especially vital to address weight stigma and improve patient adherence to postoperative regimens.

Addressing weight stigma at both the societal and individual levels is crucial. Public health campaigns could challenge prevailing narratives that stigmatize obesity and bariatric surgery, while therapeutic interventions targeting internalized stigma could foster resilience among patients. Moreover, multidisciplinary approaches that integrate mental health professionals into bariatric care teams offer a holistic solution to the complex interplay of stigma, psychological health, and weight management.

## 9. Conclusions

The interplay of psychological, familial, and systemic factors underscores the complexity of weight management post-bariatric surgery. A comprehensive, multi-level approach that addresses stigma, enhances psychological support, and empowers patients and their families could significantly improve the long-term outcomes. Future research should focus on developing and evaluating interventions that integrate these strategies to optimize the holistic care of bariatric surgery patients.
